# Wählen in der Pandemie: Herausforderungen und Konsequenzen

**DOI:** 10.1007/s41358-021-00255-6

**Published:** 2021-04-15

**Authors:** Arndt Leininger, Aiko Wagner

**Affiliations:** 1 Technische Universität Chemnitz, Chemnitz, Deutschland; 2 Wissenschaftszentrum Berlin für Sozialforschung, Berlin, Deutschland

## Einleitung

Die Herausforderungen durch das Coronavirus erfassen alle Lebensbereiche, nicht zuletzt die Politik im „Superwahljahr“ 2021. Anders als z. B. Sportveranstaltungen können Wahlen jedoch nicht einfach abgesagt, verschoben oder ohne Zuschauer:innen durchgeführt werden. Während in Israel während des Ausbruchs der Epidemie Anfang März spezielle Wahllokale mit medizinischem Personal in Schutzkleidung für unter Quarantäne stehende Wähler:innen eingerichtet wurden, haben in den USA einige Bundesstaaten die Vorwahlen verschoben. Bei den Präsidentschaftswahlen im November kam es dann zur Nutzung der Briefwahl in einem nie dagewesenen Ausmaß, was die Bekanntgabe der offiziellen Ergebnisse um Tage verzögerte, während derer Präsident Trump die Legitimität der Auszählung angriff. Auch für die Bundestagswahl im September 2021 rechnet der Bundeswahlleiter mit neuen Rekordwerten für die Briefwahl.

In diesem Beitrag befassen wir uns mit dem Wählen während der globalen Corona-Krise. Vor welche Herausforderungen stellt die Pandemie die repräsentative Demokratie und welche Folgen ergeben sich daraus? Wir diskutieren diese Frage primär mit Blick auf etablierte Demokratien wie die Bundesrepublik anhand zweier Prämissen: Die Corona-Pandemie kann sich, erstens, direkt auf die Entscheidung der Wähler:innen auswirken und, zweitens, indirekt über die Auswirkungen auf den Wahlkampf und die Durchführung der Wahl.

Wir gehen dabei davon aus, dass unsere Überlegungen nicht nur unmittelbar für die bevorstehenden Wahlen in diesem Jahr in der Bundesrepublik relevant sind, sondern im weiteren Verlauf des 21. Jahrhunderts erneut an Relevanz gewinnen könnten, denn leider steigt die Wahrscheinlichkeit von weiteren Pandemien in der Zukunft (Gavi 2020). Aufgrund der globalen Erwärmung werden zudem neben Pandemien auch Naturkatastrophen wie Stürme, Überschwemmungen, Waldbrände und Dürren voraussichtlich sowohl in ihrer Häufigkeit als auch in ihrer Intensität zunehmen, sodass die kommenden Wahlen nicht die letzten unter Krisenbedingungen gewesen sein könnten.

## Wahlentscheidung unter dem Eindruck einer globalen Pandemie

Die Corona-Pandemie ist das dominierende Thema in Politik, Gesellschaft und Medien. Kein Tag vergeht, ohne dass uns das Thema auf den Titelseiten der Tageszeitungen oder in den Berichten der Nachrichtensendungen begegnet. Ebenso drehen sich viele Gespräche in der Familie, mit Freund:innen, Verwandten, Bekannten oder Kolleg:innen um das Thema. Somit dürfte es auch, ohne dass es dafür besonderer Kampagnenaktivitäten der Parteien bedürfte, eine große Rolle für die Wähler:innen in den dieses Jahr anstehenden Landtagswahlen und der Bundestagswahl spielen. Welche möglichen Auswirkungen kann die Corona-Pandemie also auf die Wahlen haben?

Zur Beantwortung der Frage, wie sich die Pandemie-Situation auf Wahlverhalten auswirken könnte, lohnt ein Blick auf die Literatur zu den elektoralen Implikationen externer Schocks wie Terrorismus oder Naturkatastrophen. Dabei wird einerseits auf emotionale Reaktionen – die von einem „Rally ’round the flag“-Effekt bis hin zu einem angstgetriebenen Rückzug der Unterstützung für Amtsinhaber:innen reichen – und andererseits auf rationale Reaktionen – Wähler:innen neigen nach Krisen stärker zu retrospektivem Wählen, da die Regierungsleistung, insbesondere ihr Krisenmanagement, stärker in den Blick der Wähler:innen kommt – fokussiert. Diese Literatur kann uns einige auch für die momentane Krise relevante Einsichten bieten, es ist jedoch einschränkend festzuhalten, dass sie sich auf Wahlen *nach* einer Krise bezieht. Während beziehungsweise in Erwartung großer und lang andauernder Krisen könnten jedoch Vermutungen über zukünftiges Krisenmanagement eine besonders wichtige Rolle spielen; was einen dritten neueren Erklärungsansatz darstellt.

Die Verbreitung von COVID-19 stellt in erster Linie eine Bedrohung für die Gesundheit der Bürger:innen dar. In dieser Hinsicht ist sie vergleichbar mit anderen Bedrohungen wie Krieg und Terrorismus. In der Tat erklärten US-Präsident Trump, der französische Präsident Macron und andere Staatsoberhäupter ihre Länder stünden im Krieg mit dem Virus.^1^ Politikwissenschaftler:innen haben in Zeiten internationaler Konflikte sowie bei Naturkatastrophen einen „Rally ’round the flag“-Effekt festgestellt, durch den amtierende Politiker:innen an Unterstützung gewinnen (Mueller 1970; Boittin et al. 2020). Mueller argumentiert, dass Ereignisse, die zu einem solchen Effekt führen, einerseits international sein müssen und andererseits spezifisch, dramatisch und scharf fokussiert, um die öffentliche Aufmerksamkeit zu gewinnen. Diese Kriterien treffen durchaus auf die globale Pandemie zu und so könnte sich hier ein solcher „Rally ’round the flag“-Effekt einstellen. Tatsächlich stieg in Folge der ersten Welle und damit einhergehender Gegenmaßen in vielen europäischen Ländern das Vertrauen in und die Unterstützung für die Regierungen (Bol et al. 2020; Esaiasson et al. 2020; Schraff 2021). Ob sich daraus ein positiver Effekt der COVID-19-Pandemie auf zukünftige Wahlergebnisse amtierender Regierungsparteien ableiten lässt, bleibt jedoch abzuwarten.

Andere Untersuchungen haben diese Erwartungen spezifiziert: Zum Beispiel argumentieren Berrebi und Klor (2008), dass externe militärische Bedrohungen Regierungsparteien stärken, besonders dann, wenn diese von der politischen Rechten sind. Tatsächlich sehen wir, dass unter den Parteien in der Bundesregierung vor allem die Union und weniger die SPD in den ersten Monaten der Pandemie profitierte. Allerdings stellt diese mit der Kanzlerin auch die oberste Krisenmanagerin, auf die sich ein „Rally ’round the flag“-Effekt, so es ihn denn gibt, fokussieren würde. Weiterhin könnte die Pandemie auch die radikale Rechte stärken. Im Laufe der Geschichte scheinen Krankheiten mit einer erhöhten Feindseligkeit gegenüber Außenseitern einhergegangen zu sein, die teils extreme Ausmaße annahmen wie zum Beispiel die Pogrome gegen die Juden in Europa zur Zeit der Pest (Schweitzer und Perry 2002). 2020 dokumentieren Nachrichtenberichte aus aller Welt eine Zunahme von diskriminierendem Verhalten gegenüber asiatisch aussehenden Personen im Zuge der COVID-19-Pandemie.^2^ Zumindest in Deutschland konnte die politische Rechte jedoch nicht von der Corona-Krise profitieren. In den bayerischen Kommunalwahlen im März 2020 schnitt sie in stärker betroffenen Gebieten schwächer ab (Leininger und Schaub 2020) und auch in den ersten Landtagswahlen 2021 verlor sie Stimmanteile.

Eine deutlich andere Erwartung in Bezug auf die emotionalen Reaktionen der Wähler:innen formulieren Achen und Bartels (2017). Demnach werden Wähler:innen von allgemeinen Gefühlen der (Un‑)Zufriedenheit in ihrer Wahlentscheidung beeinflusst, auch wenn die Ursachen außerhalb der Kontrolle der Regierung liegen. Sie illustrieren ihr Argument mit einer Serie von Haiangriffen, die an der Ostküste der Vereinigten Staaten im Sommer vor der Präsidentschaftswahl 1916 auftraten, was nach ihrer Ansicht zu Verlusten bei den Stimmenanteilen für den amtierenden Präsidenten Woodrow Wilson führte. Einen analogen Mechanismus beschreiben Healy et al. (2010), die zeigen, dass positive externe Ereignisse, wie zum Beispiel Siege der lokalen Sportmannschaft unmittelbar vor einer Wahl, mit einem Anstieg des Stimmenanteils der Amtsinhaber:innen einhergehen. Obwohl die konkreten empirischen Analysen nicht unumstritten sind (Fowler und Montagnes 2015; Fowler und Hall 2018), ist der zugrundeliegende psychologische Mechanismus instruktiv: Menschen übertragen zuweilen Emotionen aus einem Bereich auf die Bewertungen und Urteile in einem völlig anderen Bereich, insbesondere in komplexen und unsicheren Situationen. Die COVID-19-Pandemie ist zweifellos eine solche Situation.

Hingegen argumentieren Ashworth et al. (2018), dass Katastrophen und Krisen eine Gelegenheit für Wähler:innen bieten, etwas über die Kompetenz der Regierung zu lernen. Nach dieser Perspektive nutzen Bürger:innen Wahlen, um inkompetente Amtsinhaber:innen durch kompetentere Führungspersönlichkeiten zu ersetzen (Besley 2006). So können Naturkatastrophen als entscheidende Testfälle angesehen werden, ob Wahlen am Wähler:innenwillen ausgerichtetes Regierungshandeln sicherstellen können (Gasper und Reeves 2011; Arceneaux und Stein 2006). Kompetente Krisenreaktionen würden belohnt und schlechte Leistungen bestraft. Tatsächlich zeigen Bechtel und Hainmueller (2011), dass die Dankbarkeit für Gerhard Schröders energische Reaktion auf die Elbe-Flut 2002, welche unter anderem als ursächlich für seine erfolgreiche Wiederwahl angesehen wird, auch noch Jahre nach der Flutkatastrophe in den betroffenen Gegenden in Form höherer Stimmanteile für die SPD festgestellt werden konnte. Ob sich aus dieser Perspektive ein Vor- oder Nachteil für die regierenden Parteien ergibt, muss sich erst noch erweisen. Dies hängt wesentlich davon ab, wie das Krisenmanagement bis zur Bundestagswahl durch die Bevölkerung wahrgenommen und bewertet wird.

Während die Literatur sich bisher vor allem mit Wahlen nach einer Katastrophe befasste, argumentieren Leininger und Schaub (2020), dass Wähler:innen in sich entwickelnden oder längerfristigen Krisen wie einer Pandemie ein starkes Interesse an einer Führung haben, die sie sicher durch die Krise steuern wird, und daher vor allem prospektiv orientiert wählen. In Analysen der bayerischen Kommunalwahlen vom 15. März 2020 (kurz bevor der erste bundesweite Lockdown verhängt wurde) zeigen sie, dass die CSU in bereits von COVID-19 betroffenen Gebieten besser abschnitt als in Gebieten, die noch keine Corona-Infektion verzeichnet hatten. Der vermutete Mechanismus lautet, dass Wähler:innen in den betroffenen Gemeinden die Kommunalwahlen nutzen, um ihre lokale Regierung mit der Partei in Einklang zu bringen, die auf Länderebene an der Macht ist, um eine effektivere Katastrophenabwehr und -hilfe für ihre Gemeinde sicherzustellen. Tatsächlich hat die bisherige Forschung zu Auswirkungen von Naturkatastrophen auf die Wahlen gezeigt, dass die Wähler:innen die unterschiedliche Verantwortung verschiedener Regierungsebenen berücksichtigen können (Arceneaux und Stein 2006; Malhotra und Kuo 2008; Gasper und Reeves 2011). Aus dieser Sichtweise müsste vor allem die Union, welche die Bundesregierung anführt und zugleich an den meisten Landesregierungen beteiligt ist, profitieren.

Es lassen sich also aus der einschlägigen Literatur eine Vielzahl an unterschiedlichen und teils widerstreitenden Erwartungen ableiten. Emotionale und vorausschauende Wähler:innenreaktionen dürften dabei noch am ehesten zu Gunsten der regierenden Parteien auswirken. Ob sich dies am Wahlabend bewahrheitet, wird jedoch entscheidend von den Entwicklungen in den folgenden Monaten abhängen. Über die Zeit dürfte mit dem weiteren Voranschreiten der Pandemie und ihrer schrittweisen Bewältigung nämlich mehr und mehr die retrospektive Bewertung des Krisenmanagements der Bundes- und auch Landesregierungen an Bedeutung gewinnen. Höchstwahrscheinlich wird dies ein zentrales Thema der Bundestagswahl im September, auch wenn es für die Opposition in Krisenzeiten schwieriger ist, Kritik an der Regierung zu üben, da der Vorwurf droht, das Vertrauen in die Politik und staatliches Handeln allgemein zu beschädigen. Zudem drohen andere wichtige Themen wie die Klimakrise in den Hintergrund zu treten. Es ist also davon auszugehen, dass die Corona-Krise das bestimmende Thema der Bundestagswahl 2021 sein wird. Wie sich dies auf das Abschneiden der Parteien auswirken wird, haben die bestimmenden Akteur:innen in Regierungen und Parteien noch selbst in der Hand.

## Wahlkampf und -durchführung während einer Pandemie

Nicht nur die Wahlentscheidungen, sondern auch die Wahlen als Ganzes sind von der Corona-Pandemie betroffen. Das liegt in erster Linie daran, dass der Wahlakt eine Vielzahl menschlicher Kontakte mit sich bringt: zwischen den Wähler:innen aber auch mit den Wahlhelfer:innen. In Pandemiezeiten bergen Wahlen damit ein erhöhtes Infektionsrisiko (James 2021). Um dennoch diesem demokratischen Grundrecht zur Geltung zu verhelfen, wurden seit Ausbruch des Coronavirus verschiedene Überlegungen angestellt, wie das Infektionsrisiko bei Wahlen minimiert werden kann. Drei Ansätze lassen sich unterscheiden: die Verschiebung des Wahltermins, Maßnahmen zur Reduzierung des Infektionsrisikos bei der Präsenzwahl und die (Ausweitung der) Abwesenheits- oder Briefwahl.

Die Verschiebung von Wahlen hat zum Ziel, sie nicht in einer Hochphase, sondern in einem Wellental des Infektionsgeschehens stattfinden zu lassen. Auch wenn die Verschiebung von Wahlen unter außerordentlichen Umständen auf den ersten Blick plausibel und gerechtfertigt erscheint, so berge sie zugleich Risiken, betonen James und Alihodzic (2020). Insbesondere werde durch eine Verschiebung des Wahltermins und damit die potenzielle Verlängerung politischer Machtausübung durch die Amtsinhaber:innen die Erwartungssicherheit der Bürger:innen in das Funktionierens der zentralen Institutionen des Staates und damit gegebenenfalls die *electoral integrity* (Norris 2014) beschädigt. So sei es für die Bewahrung der Integrität und Legitimität von Wahlen zentral, bei allen Entscheidungen, die Teile des Wahlzyklus betreffen – die Vorwahlperiode, den Wahlkampf, den Wahltag selbst und die Nachwahlperiode –, auf Transparenz und die Inklusion unterschiedlicher Interessen zu achten (James und Alihodzic 2020). Daher sei, wenn irgend möglich, der Einführung bzw. Erweiterung von technischen Lösungsmöglichkeiten der Vorzug zu geben.

Diesen Weg sind dann auch die meisten Länder gegangen: wurden anfangs Wahlen häufig noch verschoben, sind die meisten Länder seit dem Frühsommer 2020 dazu übergegangen, Wahlen unter Ergreifung besonderer Schutzmaßnahmen planmäßig abzuhalten (Asplund et al. 2021a). Zu diesen Maßnahmen zählen das Abstandhalten und die Personenzahlbegrenzung im Wahllokal, Handschuh- und/oder Maskenpflicht sowie -angebote im Wahllokal, Fiebermessen vor dem Einlass, Bereitstellen von Desinfektionsmitteln, Belüftung und Desinfektion des Wahllokals und der Stifte, Einstellen von Security-Mitarbeiter:innen, die die Maßnahmen überwachen und eine Einlasskontrolle vornehmen, die Erhöhung der Anzahl der Wahllokale und die Verlängerung der Öffnungszeiten (teils auf mehrere Tage), gesonderte Einlassorte für besonders vulnerable Gruppen, Werbemaßnahmen für die Tracing-App-Nutzung besonders am Wahltag sowie das Suchen von Wahlhelfer:innen explizit außerhalb von Risikogruppen (Asplund et al. 2021a).

Trotz all dieser Maßnahmen gibt es Grund zur Annahme, dass einige Bürger:innen aus Angst vor Infektionen der Wahl fernbleiben könnten. Immerhin gaben fast die Hälfte aller Nichtwähler:innen in Brasilien dies als Grund für ihre Nichtteilnahme an der Wahl an (Tarouco 2021). Die Auswirkungen der Pandemie auf die Wahlbeteiligung zeigen sich auch in einer von Noury et al. (2021) durchgeführten Studie zur Kommunalwahl in Frankreich im März 2020. Vor dem Hintergrund einer insgesamt historisch niedrigen Wahlbeteiligung wirkte sich die Nähe zu COVID-19 Clustern negativ auf die Wahlbeteiligung aus. Auch in Deutschland sank die Wahlbeteiligung in den ersten Landtagswahlen des Jahres 2021, in Baden-Württemberg und Rheinland-Pfalz, gegenüber den vorherigen Wahlen.

Die Alternative zur Verschiebung der Wahl und zu den verstärkten Hygienemaßnahmen, die zudem das Infektionsrisiko beim Wahlakt drastischer reduziert, ist die Briefwahl. Entsprechend wird auch für die Wahlen in Deutschland ein höherer Briefwahlanteil erwartet, der über den in den letzten Jahrzehnten beobachtbaren Anstieg der Briefwahlnutzung hinausgeht. Beides war bei den Landtagswahlen im März 2021 sowie bereits bei den bayerischen Kommunalwahlen am 15. März 2020, also zu Beginn der Pandemie, zu beobachten.

Bekannterweise nutzen bislang nicht alle Bevölkerungsgruppen die Briefwahl in gleichem Ausmaß. In Westdeutschland ist sie populärer als in Ostdeutschland, überproportional wird sie zudem durch Renter:innen, Schüler:innen, Studierende sowie durch Selbstständige genutzt (Lichteblau und Wagner 2019). Jedoch zeigt sich, dass die Gründe, aus denen eine Person für eine bestimmte Partei votiert, sich nicht zwischen Präsenz- und Briefwähler:innen unterscheiden (Wagner und Lichteblau 2020). Durch mehr Briefwahl oder gar eine reine Briefwahl ist daher keine Änderung des Wahlergebnisses zu erwarten (Thompson et al. 2020). Auch für die durch den ehemaligen US-Präsidenten oder AfD-Politiker:innen vorgebrachten Befürchtungen, Briefwahlen seien in quantitativ relevantem Maße fälschungsanfällig, finden sich bislang keine Belege (Eggers et al. 2021). Allerdings bringt die Briefwahl andere Probleme mit sich. So werden Briefwahlstimmen früher abgegeben, weshalb Entwicklungen und Informationen der letzten Tage des Wahlkampfes nicht für die Entscheidungsfindung berücksichtigt werden können. Noch wichtiger sind die Wahlrechtsgrundsätze der geheimen und freien Wahl (Art. 38 GG), welche am heimischen Küchentisch nicht in gleichem Maße zu gewährleisten sind wie in der Wahlkabine.

Neben diesen Implikationen für die Durchführung der Wahl hat die Pandemie auch Auswirkungen auf den Wahlkampf. Wahlkämpfe sind in normaleren Zeiten die Gelegenheiten für politische Parteien und Kandidat:innen, sich und ihre Programmatik bekannt zu machen und zu bewerben. Sie sind der Moment besonders intensiver Diskurse über die Zukunft des Landes und ermöglichen so den Bürger:innen die Meinungsbildung und Präferenzfindung. Aber in Pandemiezeiten besteht die Gefahr, dass Wahlkämpfe nicht nur politische Ideen verbreiten, sondern auch das COVID-Virus (Asplund et al. 2021b). Entsprechend führten von 51 Ländern, in denen 2020 gewählt wurde, etwa die Hälfte Beschränkungen des Wahlkampfs ein: Von der Begrenzung der Anzahl der Teilnehmer:innen bis hin zum kompletten Verbot von Wahlkampfveranstaltungen und Einschränkungen der Versammlungsfreiheit. Wo dies nicht geschah und die physische Nähe nicht eingeschränkt wurde, wurden Häufungen von Erkrankungen von Kandidat:innen und Bürger:innen beobachtet (für den brasilianischen Fall siehe Tarouco 2021). Modellierungen für 18 US-Wahlkampfveranstaltungen der Republikaner ergaben, dass sich in den folgenden zehn Wochen in den jeweiligen Wahlkreisen die Corona-Fallzahlen um 250 pro 100.000 Einwohner:innen erhöhten (Bernheim et al. 2020).

Entsprechend werden die klassischen Wahlkampfinstrumente wie z. B. der Tür-zu-Tür-Wahlkampf oder Großveranstaltungen auf Marktplätzen gegenwärtig mit Skepsis gesehen und die Rolle von Massenmedien und *social media* wächst (Wagner 2020). Die ohnehin große Rolle indirekter im Gegensatz zu direkter Kampagneninformationen (Schoen 2014) dürfte dadurch nochmals größer werden. Parteien, die über eine stark überzeugte Kernwähler:innenschaft verfügen, die nicht erst überzeugt werden muss, könnten daher gegenüber den Parteien, die stärker auf die Mobilisierung Unentschlossener setzen (müssen), profitieren. Gleichzeitig kommt den Regierungsparteien mehr öffentliche Aufmerksamkeit zu. Ob dies ein Vorteil ist, wird wesentlich von der Tonalität der Berichterstattung abhängen. Von der zu erwartenden verstärkten Nutzung der Briefwahl dürften jedoch keine starken Auswirkungen auf das Wahlergebnis ausgehen. Denn anders als in den USA, wird in Deutschland von Wähler:innen aller Parteien, mit Ausnahme vielleicht der AfD, die Pandemie als reale und ernstzunehmende Bedrohung wahrgenommen bei gleichzeitigem hohen Vertrauen in eine ordnungsgemäße Wahl.

## Fazit

Dass die Corona-Pandemie das bestimmende Thema der Wahlen in der Bundesrepublik und darüber hinaus ist, steht außer Frage. Als Krise kann sie unterschiedliche Reaktionsweisen seitens der Bürger:innen evozieren: Erstens einen „Rally ’round the flag“-Effekt, nach dem sich Wähler:innen verstärkt hinter die Amtsinhaber:innen stellen, zweitens eine Zunahme retrospektiven, leistungsbewertenden Wählens, nach welchem die Wähler:innen vor allem das Verhalten der politischen Akteure während der Krise beurteilen und auf dieser Basis ihre Wahlentscheidungen fällen oder drittens stärkeres prospektives und auf parteipolitische Homogenität hin orientiertes Wählen, also die Überlegungen, dass bei Amtsführung der gleichen Partei(en) auf unterschiedlichen Ebenen sich Vorteile ergeben. Während im ersten Fall die Regierungsparteien profitierten, ist der Einfluss auf Wahlergebnisse im zweiten Fall abhängig von (der Bewertung) der Bearbeitung der Krise. Bürger:innen, die mit dem Krisenmanagement unzufrieden waren, dürften vermehrt den Oppositionsparteien ihre Stimme geben, Zufriedene machten demnach ihr Kreuz eher bei den Parteien der Großen Koalition. Nach der dritten Entscheidungslogik sollten ebenfalls Union und SPD als die Parteien mit den meisten Regierungsbeteiligungen in Bund und Ländern profitieren.

Auch die Auswirkungen auf die Wahlkampfführung sind nicht für alle Parteien gleich. Verminderte Kontaktmöglichkeiten und geringere Wahlbeteiligungsraten schaden eher jenen Parteien, die stärker als andere auf die Mobilisierung ihrer Anhänger:innenschaft setzen (müssen). Zudem kann erwartet werden, dass Oppositionsparteien mit verminderten Möglichkeiten der Sichtbarwerdung im Wahlkampf tendenziell vor größere Probleme gestellt sein werden als die allein schon durch die exekutiv-lastige Krisenpolitik sichtbareren Regierungsparteien. Parteipolitisch neutral dagegen sollten die Pandemieeffekte auf die Organisation der Präsenzwahl sowie den Modus der Stimmabgabe ausfallen – weder die Gesundheitsschutzmaßnahmen in den Wahllokalen noch die zu erwartende vermehrte Briefwahlnutzung lassen Gewinne nur für bestimmte Parteien erwarten.

Insgesamt legen die präsentierten Überlegungen ebenso wie die bisherigen komparativen Befunde einen optimistischen Schluss nahe für die Möglichkeiten funktionierender, integrer und damit die demokratische Legitimität stützender Wahlen in etablierten Demokratien wie der Bundesrepublik auch im Pandemiejahr 2021.

## Footnotes

1 US-Präsident Donald Trump machte solche Bemerkungen bei Pressegesprächen (https://time.com/5806657/donald-trump-coronavirus-war-china/, abgerufen am 04.08.2020), während Macron in einer Fernsehansprache mehrfach von einem Krieg gegen das Virus sprach (https://www.politico.eu/article/emmanuel-macron-on-coronavirus-were-at-war/, abgerufen am 04.08.2020).

2 Siehe z. B. den folgenden Artikel der New York Times vom 23. März 2020 (https://www.nytimes.com/2020/03/23/us/chinese-coronavirus-racist-attacks.html) und Tabri et al. (2020).
